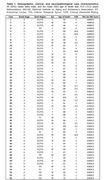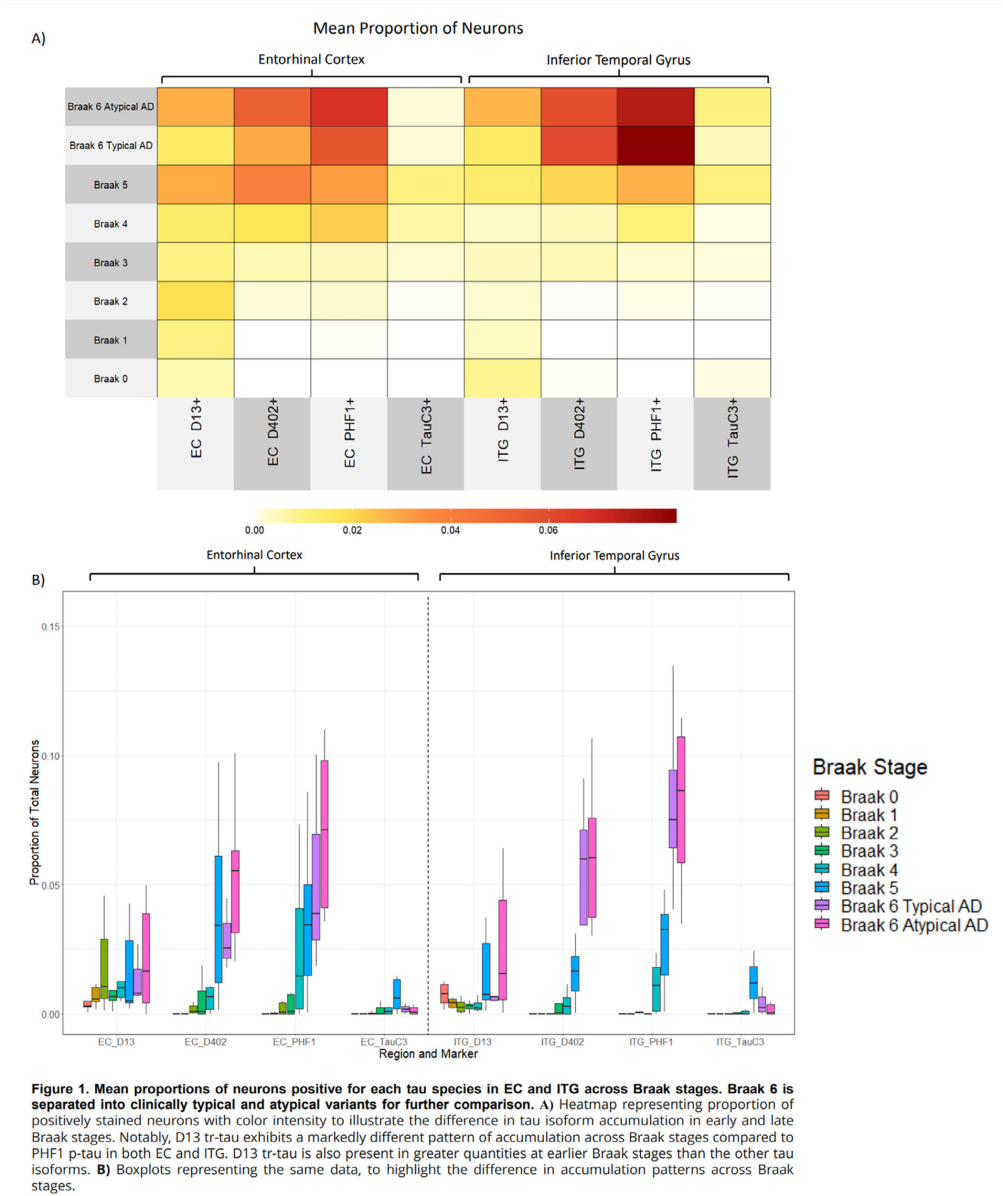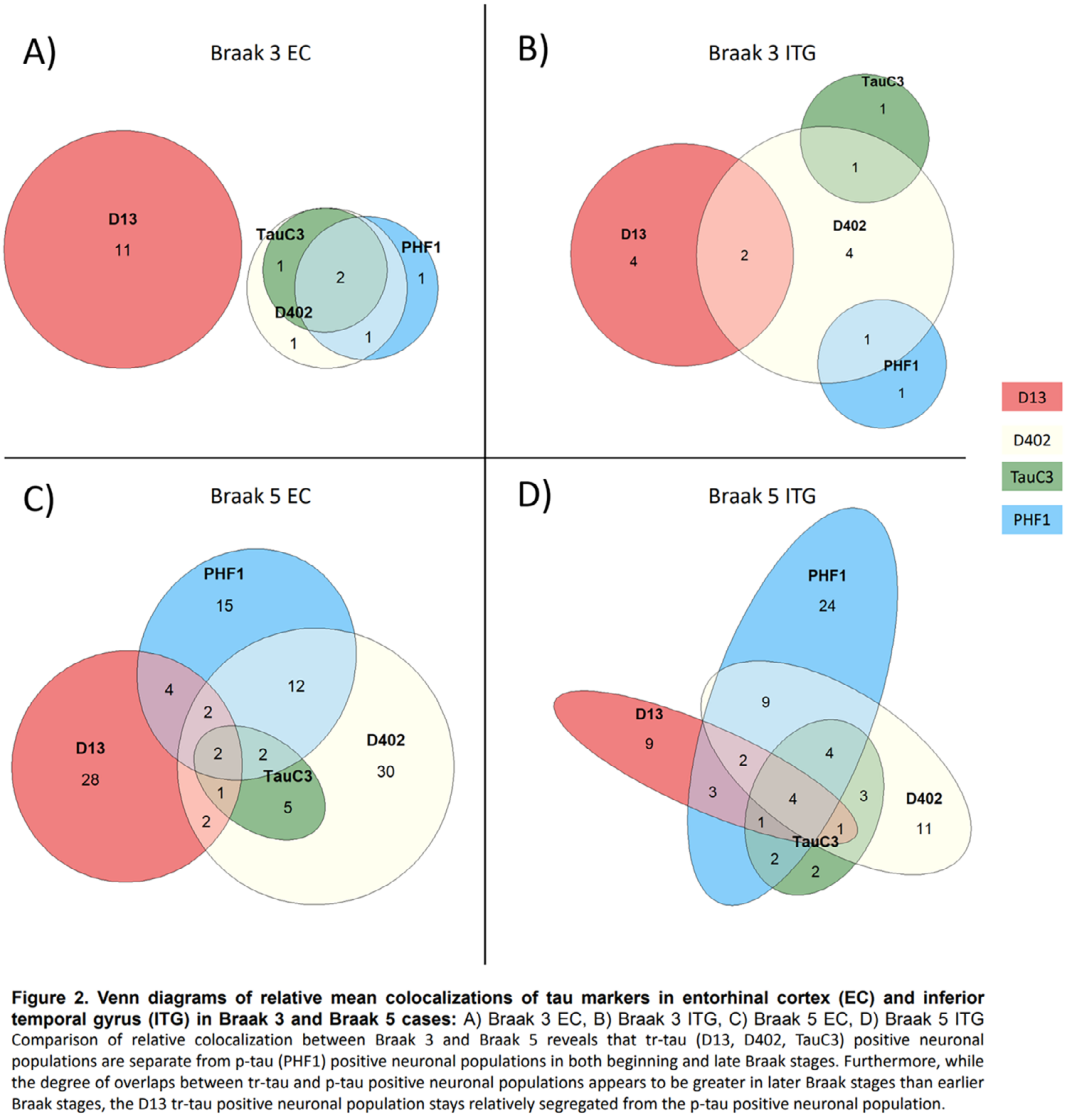# Truncated tau accumulates before hyperphosphorylated tau and in relatively distinct neuronal subpopulations in entorhinal cortex and inferior temporal gyrus in Alzheimer’s disease patients

**DOI:** 10.1002/alz.093137

**Published:** 2025-01-03

**Authors:** Ian Michael Oh, Song Hua Li, Felipe Luiz Pereira, Prabhleen Kaur, Anya Raju, Rushil Jerfy, Claudia Kimie Suemoto, Renata Elaine Paraizo Leite, Vitor Ribeiro Paes, Roberta Diehl Rodriguez, Andrew J. Ambrose, Salvatore Spina, William W. Seeley, Bruce L. Miller, Michelle R. Arkin, Lea T. Grinberg

**Affiliations:** ^1^ Memory and Aging Center, UCSF Weill Institute for Neurosciences, University of California, San Francisco, San Francisco, CA USA; ^2^ Memory and Aging Center, UCSF Weill Institute for Neurosciences, University of California San Francisco, San Francisco, CA USA; ^3^ UC Berkeley, Berkeley, CA USA; ^4^ Memory and Aging Center, Weill Institute for Neurosciences, University of California San Francisco, San Francisco, CA USA; ^5^ University of São Paulo Medical School, São Paulo, São Paulo Brazil; ^6^ Division of Geriatrics, University of São Paulo Medical School, São Paulo, São Paulo Brazil; ^7^ University of São Paulo Medical School, São Paulo Brazil; ^8^ UCSF Department of Pharmaceutical Chemistry and Small Molecule Discovery Center, University of California, San Francisco, San Francisco, CA USA; ^9^ Department of Pathology, University of California, San Francisco, San Francisco, CA USA; ^10^ Global Brain Health Institute, San Francisco, CA USA; ^11^ Department of Pathology, University of Sao Paulo Medical School, São Paulo, São Paulo Brazil; ^12^ Global Brain Health Institute, University of California San Francisco, San Francisco, CA USA; ^13^ Memory & Aging Center, Department of Neurology, University of California in San Francisco, San Francisco, CA USA; ^14^ Department of Pathology, University of California San Francisco, San Francisco, CA USA

## Abstract

**Background:**

Alzheimer’s disease (AD) features stereotypical spread of hyperphosphorylated tau (p‐tau) and beta‐amyloid. Although other pathological tau posttranslational modifications (PTMs) have been described in AD, a prevalent disease model preconizes that other tau PTMs always coincide with p‐tau, making the latter an excellent marker of pathological tau burden. We showed in experimental studies that truncated tau (tr‐tau), a pathological tau PTM generated via cleavage by active caspases, is as common as p‐tau in neurons at late AD stages; however, only about 40% of tr‐tau positive neurons also show p‐tau positivity. This makes tr‐tau a potential AD marker previously invisible at neuropathological investigation or potential target of diagnostic tool development. We sought to determine how early in AD tr‐tau is detected and to what degree tr‐tau and p‐tau neuronal populations overlap at early AD stages.

**Method:**

Our analysis included 56 cases (Table 1) from across all AD Braak stages (BB = 0‐6; N = 56). We used multiplex immunofluorescence to probe tr‐tau (D13, D402, TauC3) and p‐tau (PHF1) species in the same tissue slides of postmortem human brain tissue. We then quantified neuronal tau pathology and colocalization in images of entorhinal cortex (EC) and inferior temporal gyrus (ITG) as areas representative of p‐tau pathology in intermediate AD stages.

**Result:**

We detected D13 tr‐tau in lower BB and in greater quantities than p‐tau at low BB (Fig. 1A). The EC accumulated more D13 tr‐tau than the ITG in early BB (Fig. 1B). Overlap between D13 tr‐tau and p‐tau was minimal in both regions from early to late BB, with 0% at BB = 3 in both regions and 16.7% and 16.1% at BB = 5 in EC and ITG, respectively (Fig. 2).

**Conclusion:**

Neuronal D13 tr‐tau deposits develop in AD before p‐tau pathology. Across AD Braak stages, the % of overlapping between D13 tr‐tau and p‐tau positive neuronal populations is moderate at best; these results suggest neuronal accumulation of D13 tr‐tau occurs in a selectively vulnerable manner. This corroborates D13 tr‐tau as a potential biomarker of tau pathology in AD overlooked by p‐tau screening. Future plans include analysis of these pathological markers between clinically typical and atypical variants of AD.